# RNA Immune Signatures from Pan-Cancer Analysis Are Prognostic for High-Grade Serous Ovarian Cancer and Other Female Cancers

**DOI:** 10.3390/cancers12030620

**Published:** 2020-03-07

**Authors:** Wendell D. Jones, Chad M. Michener, Charles Biscotti, Iona Braicu, Jalid Sehouli, Mahrukh K. Ganapathi, Ram N. Ganapathi

**Affiliations:** 1 Bioinformatics Group, Q^2^ Solutions - EA Genomics, 5927 S Miami Blvd, Morrisville, NC 27560, USA; wendell.jones@q2labsolutions.com; 2 Division of Gynecologic Oncology, Cleveland Clinic, 9500 Euclid Avenue, Cleveland, OH 44195, USA; michenc@ccf.org; 3 Department of Anatomic Pathology, Cleveland Clinic, 9500 Euclid Avenue, Cleveland, OH 44195, USA; bicotc@ccf.org; 4 Department of Gynecology, Charité Medical University of Berlin, Augustenburger Platz 1, 13353 Berlin, Germany; elena.braicu@charite.de (I.B.); jalid.sehouli@charite.de (J.S.); 5 Department of Cancer Pharmacology, Levine Cancer Institute, Carolinas Medical Center, 1021 Morehead Medical Drive, Charlotte, NC 28204, USA; mahrukh.ganapathi@carolinas.org

**Keywords:** RNA immune signature, tumor microenvironment, high-grade serous ovarian cancer, survival outcome, multivariable Cox models

## Abstract

Immune cell infiltrates within the tumor microenvironment can influence treatment response and outcome in several cancers. In this study, we developed RNA-based immune signatures from pan-cancer analysis that could serve as potential markers across tumor types and tested them for association with outcome in high-grade serous ovarian cancer (HGSOC) and other female cancers. Pan-cancer RNA-Seq cluster analysis of immune-related gene expression profiles in The Cancer Genome Atlas (TCGA) from 29 different solid tumors (4446 specimens) identified distinct but concordant gene signatures. Among these immune signatures, Cytotoxic Lymphocyte Immune Signature (CLIS), T-cell trafficking (TCT), and the TCT to M2 tumor-associated macrophage (M2TAM) ratio (TCT:M2TAM) were significantly (*p* < 0.05) associated with overall survival (OS), using multivariable Cox proportional hazards regression models, in a discovery cohort and two independent validation cohorts of HGSOC patients. Notably, the TCT:M2TAM ratio was highly significant (*p* ≤ 0.000001) in two HGSOC cohorts. Immune signatures were also significant (*p* < 0.05) in the presence of tumor cytoreduction, *BRCA1/2* mutation, and *COL2A1* expression. Importantly, the CLIS and TCT signatures were also validated for prognostic significance (*p* < 0.05) in TCGA cohorts for endometrial and high tumor mutational burden (Hi-TMB) breast cancer. These immune signatures also have the potential for being predictive in other cancers and for patients following different treatment strategies.

## 1. Introduction

The tumor microenvironment (TME) plays an important role in tumor growth and treatment response. A key component of the TME involves immune cell infiltrates that orchestrate the innate and adaptive immune response. The interaction of immune cells with tumor cells can lead to tumor eradication or progression depending on the type and activity of immune cells that are present in the TME. Studies of the molecular mechanisms involved in immune surveillance within tumors has led to development of promising immunotherapies that have benefited a subset of patients. Furthermore, the immune landscape can also influence response to chemotherapy.

High-grade serous ovarian cancer (HGSOC), the most common subtype of epithelial ovarian cancer (EOC), is usually diagnosed at an advanced stage [1]. Although most patients respond to initial adjuvant platinum/taxane-based chemotherapy, the rate of recurrence is relatively high [1,2]. Several clinical, genomic, and immune factors have been correlated with long-term survival, including age, stage, tumor grade, optimal or sub-optimal primary cytoreductive surgery, homologous-recombination deficiency (HRD), *BRCA1/BRCA2* mutations, and the presence of intra-tumoral immune cells [3,4,5,6,7,8,9,10]. Patients with high levels of CD8^+^ tumor infiltrating lymphocytes, as assessed by immunohistochemistry [11,12,13], and patients with high ratios of M1 macrophage cells to M2 tumor-associated macrophage (M2TAM) cells in the TME [14] exhibit improved survival. Further, *CXCR3*, and its corresponding ligands (*CXCL9*, *CXCL10*, and *CXCL11*), which are frequently expressed by M1 macrophage and other cells in the TME and play a major role in T cell trafficking [15,16,17,18,19,20], are also associated with overall survival (OS) in EOC [21,22].

**Figure 1:**
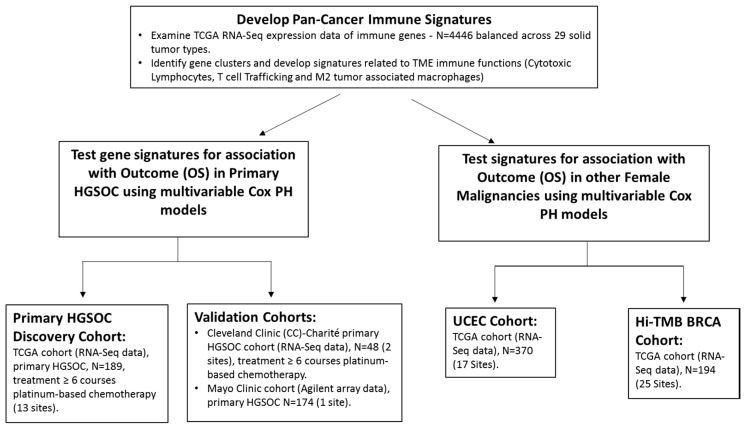
Study design for development of pan cancer immune signature from 29 different solid tumor types in The Cancer Genome Atlas (TCGA) and testing of the immune signatures for relationship to outcome in HGSOC and other female cancers. Patients in all high-grade serous ovarian cancer (HGSOC) cohorts underwent the cytoreductive surgery of a primary tumor followed by adjuvant platinum-based chemotherapy. OS (overall survival); proportional hazards (PH) UCEC (uterine corpus endometrial cancer); breast cancer (BRCA); tumor mutational burden (TMB); overall survival (OS).

The relationship between immune status determined by gene expression-based biomarkers, treatment response, and OS for EOC patients is indeed complex, since no significant association between published expression-based immune signatures and OS was reported using a univariable Cox model in a cohort of 260 patients from TCGA [8,21]. Konecny et al. [22] developed a distinct RNA-based immune response signature for EOC mimicking the immunoreactive subtype from TCGA and found it was significantly associated with OS in two independent cohorts, but not in TCGA EOC dataset. In this study, using the experimental approach outlined in Figure 1, we conducted pan-cancer analysis from 29 different solid tumors (4446 specimens) in TCGA to develop immune signatures that could be applied in a clinical setting to several different cancers, including EOC.

## 2. Results

### 2.1. Pan-Cancer Analysis of TCGA RNA-Seq Data

**Figure 2:**
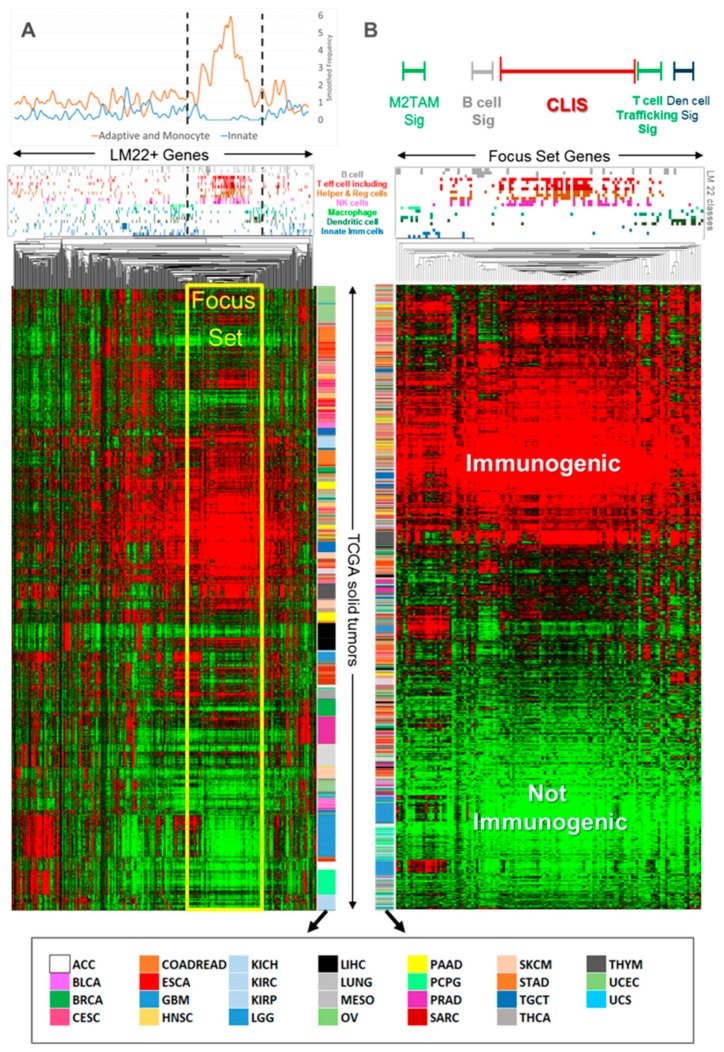
Pan-cancer immunome-clustered heat map of immune-related genes from a stratified uniform sampling of 4446 solid tumor samples. Each tumor specimen is a row and each immunome gene is a column in each heatmap. Each gene is a member of one or more of the immunome 22 classes, where class membership is indicated in the color-coded graph immediately above each heat map. (**A**) denotes a hierarchical clustering where each gene is centered by column. (**B**) denotes the re-clustering of TCGA solid tumor samples using the focus set of 126 genes and annotation of the immune signatures of interest. Abbreviations: CLIS, Cytotoxic Lymphocyte Immune Signature; Den, dendritic; Sig, signature; TAM, tumor associated macrophage. A detailed description of each gene class and its color code is provided in Figure S1. An explanation of TCGA codes is provided in Table S1.

A heatmap (Figure 2A) of the pan-cancer analysis of genes expressed in immune cells in TCGA solid tumors showed that both gene–cancer type interactions and cancer-independent characteristics were seen in the clustered expression profile. Various related subsets of genes appeared to express in a similar coordinated fashion across many tumor types and specimens. The re-clustering of a gene focus set from Figure 2A of 126 primarily adaptive and monocyte-related genes appeared to cluster into distinguishable immune compartments (Figure 2B with key to color codes shown in Figure S1). In contrast to Figure 2A, the gene focus set in Figure 2B showed relatively little cancer-specific clustering.

Several important pan-cancer features were observed from the cluster analysis (Figure 2B). Tumors mostly segregated into immune-active and immune-silent groups. Immune-active tumors did not generally cluster by cancer type and high levels of adaptive immune activity were seen in all tumor types. Exceptions were observed for glioblastoma multiforme (GBM) and lower grade glioma (LGG), which exhibited poor immunogenic activity. Several immune subsystems of co-expressing genes that are potentially useful as immune signatures were identified, including a wider class of cytotoxic lymphocytes represented by 57 genes that included CD8 T effector, natural killer (NK), and T helper cells; T cell Trafficking (TCT) is represented by three genes (associated with the *CXCR3* ligands, *CXCL9 CXCL10*, *CXCL11*); and M2TAM is represented by four genes.

### 2.2. Association of Pan-Cancer Derived Immune Signatures with Survival in HGSOC

**Figure 3:**
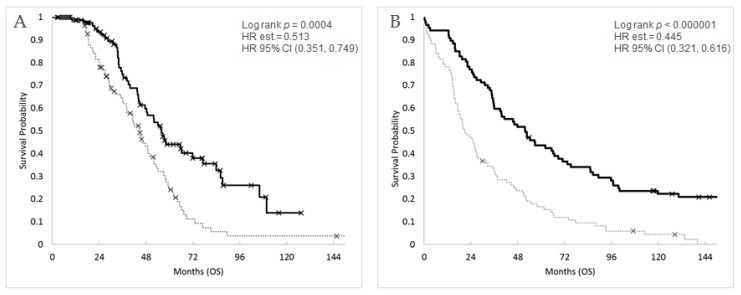
Kaplan–Meier plots of high (solid) vs. low (dotted) values of the T cell trafficking:M2 tumor-associated macrophages—TCT:M2TAM (ratio) signature in tumors from high-grade serous ovarian cancer (HGSOC) patients from (**A**) TCGA cohort (OS, *n* = 189) and (**B**) the Mayo Clinic cohort (OS, *n* = 174). High vs. low is determined by the median of TCT:M2TAM for the cohort. Censored overall survival (OS) times are represented by an X. HR (hazard ratio) and CI (confidence interval).

Table 1 shows the patient characteristics of three independent HGSOC cohorts used for testing the derived immune signatures. The Cleveland Clinic-Charité cohort is enriched for long-term survivors as a percentage relative to the other cohorts (42% versus 21–29%). The immune signatures derived from the pan-cancer analysis were first tested in the HGSOC TCGA discovery cohort. The results (Table 2 and Figure S2) reveal that the cytotoxic lymphocyte immune signature (CLIS), TCT, and the TCT to M2TAM ratio (TCT:M2TAM), in the presence of age, stage, and cytoreductive surgery, were significantly associated with OS: CLIS, (*p* = 0.038; HR = 0.807, 95% CI [0.659,0.989]); TCT, (*p* = 0.014; HR = 0.795, 95% CI [0.662,0.954]); and TCT:M2TAM (*p* < 0.000001; HR = 0.603, 95% CI [0.482,0.754]). This is the first time, to the best of our knowledge, that any RNA-based immune signature has demonstrated association with OS endpoints in TCGA HGSOC dataset. Validation of the defined signatures in two other HGSOC cohorts (Cleveland Clinic-Charité and Mayo Clinic) revealed that CLIS and TCT were significantly associated with OS (*p* < 0.05) in the presence of age and stage with similar HR estimates (Table 2 and Figure S2). Contrary to several other studies, primary surgical cytoreduction (optimal or sub-optimal) was not found to be significant by itself or in the presence of various factors, including immune status, in association with OS in TCGA cohort. As a result, primary cytoreduction was not included in the multi-variable Cox PH models for TCGA. Primary surgical cytoreduction (optimal or sub-optimal) was highly significant as a co-variable in the Mayo Clinic cohort (*p* < 0.00001) and had mixed significance (*p* = 0.014 to 0.067) in the Cleveland Clinic-Charité cohort. These results indicated that higher levels of cytotoxic lymphocytes and TCT were associated with reduced risk and better outcomes (HR < 1). Interestingly, the TCT:M2TAM signature, which was highly significant for TCGA and the Mayo Clinic cohort, exhibited *p*-values that were two to four orders of magnitude more significant than for the individual signatures (Table 2 and Figure 3). In contrast, *p*-values for the M2TAM immune signature in particular were not significantly associated with OS either individually or with other co-variables and would not have initially suggested that M2TAM would be so highly associated with OS when combined with TCT as a ratio.

### 2.3. Association of the Immune Signatures in the Presence of other Cofactors

Beyond their prognostic value, we investigated whether the derived immune signatures were significantly associated with disease-free survival (DFS) in the presence of other known predictive biomarkers, e.g., *BRCA1/2* somatic mutational status [9,10] and *COL2A1* expression [23]. In TCGA, *BRCA1/2* somatic mutational status was significantly associated with DFS in the presence of age, stage and each RNA-based immune signature (Table S2). The statistical significance and HR estimates for each immune signature were not substantially impacted by the presence of *BRCA1/2* somatic mutational status. This analysis was not carried out for the other HGSOC cohorts as *BRCA1/2* mutational status was not available. In the TCGA and Cleveland Clinic-Charité DFS cohorts, *COL2A1* expression was jointly significant with the immune factors CLIS and TCT in the presence of the other clinical factors. In fact, the statistical significance of the immune factors increased and HR estimates were stable for CLIS and TCT when *COL2A1* expression was added to the multivariable PH model (Table S3). Thus, immune status is important in explaining variation in DFS and OS across multiple cohorts even when patient age, tumor stage, *BRCA1/2* somatic mutational status, tumor cytoreduction, and *COL2A1* expression is known.

### 2.4. Significance of Pan-Cancer-Based Immune Signature in other Cancers

To test the applicability of the study-derived immune signatures in other cancers, we probed TCGA RNA-Seq data for uterine corpus endometrial cancer (UCEC) and high tumor mutational burden (Hi-TMB) breast cancer (BRCA) patients [24,25]. The patient characteristics for these two cohorts are shown in Table S4. Similar to HGSOC, the immune signatures for CLIS (*p* = 0.001 in UCEC and *p* = 0.002 in Hi-TMB breast cancer) and TCT (*p* = 0.036 in UCEC and *p* = 0.032 in Hi-TMB breast cancer) were significantly associated with outcome in multivariable Cox PH models (Table 3) suggesting the potential utility of the immune signatures for other female cancers. Hi-TMB breast cancer OS multivariable Cox PH models also included progesterone receptor (PR) status, since progesterone is a known predictor of outcomes in breast cancer and was significantly associated with OS with each immune signature.

## 3. Discussion

Cytotoxic T lymphocytes play a significant role in the tumor cytotoxic response. However, different immune components within the TME can collectively impact tumor growth characteristics. Several studies have characterized immune subtypes based on the RNA expression of immune-related genes, but the clinical value of these gene signatures has not been reliably described among different patient cohorts. Therefore, the establishment of pan-cancer RNA-based immune signatures with broad predictive and/or prognostic applicability for different tumor types would be helpful in guiding treatment strategies

Using a pan-cancer approach of solid tumors, we derived RNA-based immune-gene signatures that represented different immune activities and successfully tested the prognostic value of three important signatures in different tumors. These signatures included CLIS, TCT, and TCT:M2TAM, representing the ratio of two plastic immune activities that exert divergent effects on tumor progression (tumor inhibitory vs. tumor promoting, respectively). Our results demonstrate that CLIS, TCT, and TCT:M2TAM signatures were significantly associated with OS in the TCGA HGSOC discovery cohort. The robustness of these signatures was confirmed in two validation cohorts of HGSOC. In addition, CLIS and TCT signatures had a value beyond HGSOC, showing significance with the OS of UCEC and Hi-TMB breast cancer patients. Thus, CLIS and TCT were significantly associated with OS in all patient cohorts tested, independent of the RNA measurement platform (RNA-Seq or microarray) and in the presence of several important co-variables.

Interestingly, the TCT:M2TAM activity was shown to be a more significant prognostic indicator than individual signatures in TCGA and the Mayo Clinic HGSOC cohorts. The improved HR and significance of the TCT:M2TAM in the larger TCGA and Mayo Clinic cohorts as well as the similar directionality of the HR and low *p* value (0.067) for the smaller Cleveland Clinic-Charité cohort illustrate the potential for combining immune regulatory activity with anti-tumor immune activity to explain a larger portion of the variation observed in outcomes at least for HGSOC. Indeed, previous studies have shown that tumor progression can be influenced by the differential expression of M1 and M2 macrophages [26]. It has also been reported that the depletion of tumor-associated macrophages can improve T cell migration [27] and that the repolarization of M2-to-M1 macrophage within the TME using a supramolecule improves anti-tumor effects in pre-clinical models of breast cancer and melanoma [28]. Thus, our results suggest that immune activity is not unidimensional relative to outcomes and that a more complete view of the TME is needed to provide insights for improving patient outcomes with novel chemotherapy and/or immunotherapy strategies.

Our results demonstrating the association of immune signatures with the adjuvant-chemotherapy-response is supported by recent studies demonstrating the augmentation of a pre-existing immune response in HGSOC patients treated with neo-adjuvant chemotherapy [29,30]. Furthermore, our results reveal that the significance of CLIS and TCT was maintained when other predictive markers of HGSOC, *BRCA1/2* mutational status [3], primary cytoreduction [5], or *COL2A1* expression [23] were included in multivariable Cox PH models for predicting DFS in HGSOC cohorts.

In addition to being prognostic for HGSOC, CLIS and TCT signatures were also prognostic for other female malignancies, UCEC and Hi-TMB breast cancer. The strength of the association for the different cancer types evaluated was greatest when immune activity was measured on the continuum rather than as High/Low, implying that risk was proportional to levels of CLIS and TCT. Interestingly, CLIS showed consistent HR estimates to those reported for CD8+ T cells measured by immunohistochemistry [7] and CLIS, TCT, and TCT:M2TAM were more significantly associated with survival in TCGA HGSOC cohort than other reported [31,32] immune signatures.

Immunotherapy has made substantial progress in treating several cancers [13,33,34,35]. However, the absence of suitable biomarkers has made it difficult to identify patients that would benefit from immunotherapy treatment. The pan-cancer immune signatures defined in this study that were representative of both the adaptive and innate/inflammatory immune tumor microenvironment and predictive of outcome to chemotherapy may also be useful in guiding treatment decisions for selecting patients being considered for immunotherapy. The predictive capabilities of inflammatory/immunosuppressive immune signatures suggest consideration of these signatures in addition to the routinely employed biomarkers, e.g., PD1/PDL1 expression for immunotherapy treatment protocols.

## 4. Materials and Methods

### 4.1. Pan-Cancer RNA-Seq Data Analysis for Derivation of Immune Signatures

The experimental approach is outlined in Figure 1. RNA-Seq profiles in TCGA from 29 different solid tumor types (Table S1) were evaluated to identify patterns of gene co-expression associated with major immune subsystems. A stratified random sampling approach was employed to examine 4446 pan-cancer tumor specimens. Up to 200 specimens from each type were randomly selected to prevent bias in statistical analysis.

For derivation of the immune signatures, normalized TCGA expression data were mean centered by gene and hierarchically clustered (centroid-based) by gene and tumor specimens using correlation as a measure of similarity. The immunome model was based on the LM22 classification of > 500 genes [36] supplemented with additional genes from other studies [37,38]. All immune signatures were derived while blinded to clinical outcome information.

### 4.2. Patient Cohorts

The applicability of the immune signatures was tested in TCGA RNA-Seq HGSOC dataset (discovery cohort), *n* = 189 [8] as the prototypical tumor type and validated in two other HGSOC patient cohorts, Cleveland Clinic, and Charité Medical University of Berlin (*n* = 48), and Mayo Clinic, *n* = 174 [22], as well as TCGA uterine corpus endometrial cancer (UCEC) cohort (*n* = 370) and a subset of TCGA breast cancer (BRCA) cohort (*n* = 194) that consisted of patients with high TMB. Hi-TMB was defined as TMB > 2 nonsynonymous somatic variants per Mb. In TCGA and Cleveland Clinic-Charité HGSOC cohorts we selected patients who had cytoreductive surgery followed by ≥ 6 courses of adjuvant platinum-based chemotherapy. For the Mayo Clinic patients, all patients received adjuvant platinum-based chemotherapy. Ethics approval and consent to participate. For our study cohort (also known as the Cleveland Clinic-Charité cohort), tumor specimens were obtained at cytoreductive surgery according to institutional review board approved protocols (IRB 4614), with fully informed consent. The study was performed in accordance with the Declaration of Helsinki.

### 4.3. RNA-Seq and Microarray Analysis

The RNA-Seq normalized count data for TCGA cohorts was downloaded from TCGA repositories within dbGAP (now part of the Cancer Genomics Cloud). The RNA-Seq data was aligned to the genome (hg19) using Mapsplice, quantified relative to the transcriptome using RNA-Seq by expectation-maximization (RSEM) [39], and consolidated to the gene level. Counts were further normalized using upper quartile methods across generally detected genes to be more compatible with the discovery cohort.

Tumor specimens for the Cleveland Clinic/Charité cohort were obtained at primary cytoreductive surgery from patients providing complete informed consent according to Institutional Review Board approved protocols. RNA samples were prepped using 100 ng total RNA input following the Illumina TruSeq Total RNA prep protocol and were sequenced on Illumina HiSeq flow cells in a 50-b paired-end fashion with each sample having a minimum of 50 M clusters (paired reads). The same bioinformatic methods used for TCGA RNA-Seq were applied except that alignment was performed using STAR v2.4.

RNA expression data for the Mayo Clinic (Agilent array-based) cohort were extracted from the Gene Expression Omnibus (GEO) file GSE53963_series_matrix.txt [22]. When multiple probes existed for a gene, the probe with the greatest detection level across the cohort was preferentially selected.

### 4.4. Statistical and Survival Analysis

All immune signatures used a weighted average of individual gene expression values within the signature (log_2_ scale) to create a score per signature. Immune signatures were standardized (through a linear transform of log_2_ values) relative to each cohort so that a one-unit change in an immune signature score implied an approximate one standard deviation change within the study population. The log_2_ signature values were linearly transformed so that the 15th and 85th percentile values were recoded to −1 and 1, respectively to compare hazard ratio (HR) estimates between studies and signatures.

For survival analysis, Cox proportional hazards (PH) regression models were implemented using PROC PHREG from SAS v9.4. Confidence intervals for HR estimates used the Wald 95% limits. For this analysis, patient age, tumor stage, and various immune factors were considered simultaneously. Additionally, we considered other factors in several multivariable survival models as appropriate for either OS or DFS: for example, *BRCA1/BRCA2* somatic mutational status (mutated vs. wild-type), primary cytoreductive surgery (optimal or sub-optimal or unknown), and *COL2A1* expression (high and low) for HGSOC, and progesterone receptor (PR) status (positive vs. negative) for breast cancer. For TCGA analysis, patient age, tumor stage, grade, and primary cytoreductive surgery status are available via the Broad Firehose portal [40].

## 5. Conclusions

In conclusion, our study demonstrates convincingly that multiple RNA-based immune signatures identified using a pan-cancer approach are prognostic for OS in HGSOC as well as other female cancers. These findings also suggest that these signatures potentially have predictive/prognostic value in other solid tumors. Thus, a comprehensive understanding of the genomic differences and the association of relevant RNA-based biomarkers with immune response could significantly influence clinical decisions, including the development of novel targeted strategies and immune checkpoint blockade therapy.

## Figures and Tables

**Table 1:** Patient characteristics for high-grade serous ovarian cancer (HGSOC) cohorts.

Cohort Characteristics	TCGA	Cleveland Clinic-Charité	Mayo Clinic
No. of patients (*n*) ^a^	189	48	174
*n* with *t* ≤ 1-year (censored) ^b^	26 (24)	4 (0)	24 (0)
*n* with 1 year < *t* ≤ 5-year (censored) ^b^	123 (32)	24 (6)	99 (2)
*n* with *t* > 5- year (censored) ^b^	40 (19)	20 (14)	51 (19)
% patients w/censored survival	40%	42%	12%
Minimum % with ≥ 5-year survival	21%	42%	29%
Stage ≤ 2	12 (6%)	6 (13%)	8 (5%)
Stage 3	154 (81%)	38 (79%)	125 (72%)
Stage 4	23 (12%)	4 (8%)	41 (24%)
Primary Surgical Cytoreduction Status: Opt. – Subopt. – Unknown ^c^	130-12-47	34-14-0	128-48-3
Median Age (years)	57	61	64
Interquartile Range of Age (years)	(51, 67)	(49, 70)	(55, 72)
RNA measurement platform	RNA-Seq	RNA-Seq	Agilent

^a^ Patients in each cohort had cytoreductive surgery followed by adjuvant platinum and/or taxane-based chemotherapy. ^b^ Survival time (OS) is represented by “*t*”. ^c^ Optimal (Opt.), Suboptimal (Subopt.) and Unknown.

**Table 2:** Multivariable Cox proportional hazards (PH) model results for overall survival (OS) using immune signatures Cytotoxic Lymphocyte Immune Signature (CLIS), T cell trafficking (TCT), T cell trafficking:M2 tumor associated macrophages (TCT:M2TAM) signatures from three independent high-grade serous ovarian cancer (HGSOC) cohorts.

Multivariable. Cox PH Models ^a^	TCGA			Cleveland Clinic-Charité			Mayo Clinic		
	*p*-Value	HR ^b^ Est.	HR 95% Wald CI	*p*-Value	HR^b^ Est.	HR 95% Wald CI	*p*-Value	HR^b^ Est.	HR 95% Wald CI
**CLIS**	0.038	0.807	(0.659,0.989)	0.048	0.670	(0.451,0.996)	0.003	0.767	(0.643,0.915)
Age	0.088	1.016	(0.998,1.034)	0.286	1.018	(0.985,1.053)	0.058	1.016	(0.999,1.030)
Stage	0.659			0.019			0.006		
Primary surgical cytoreduction ^c^	Not significant			Not significant			<0.000001	0.400	(0.277,0.579)
**TCT**	0.014	0.795	(0.662,0.954)	0.014	0.587	(0.384,0.897)	0.0002	0.716	(0.601,0.852)
Age	0.112	1.015	(0.997,1.033)	0.350	1.019	(0.980,1.059)	0.074	1.014	(0.999,1.029)
Stage	0.886			0.120			0.008		
Primary surgical cytoreduction ^c^	Not significant			0.059	0.443	(0.190,1.031)	<0.000001	0.382	(0.263,0.554)
**TCT:M2TAM** ^d^	<0.000001	0.603	(0.482,0.754)	0.067	0.660	(0.422,1.030)	<0.000001	0.570	(0.458,0.710)
Age	0.228	1.011	(0.993,1.030)	0.539	1.012	(0.974,1.051)	0.073	1.013	(0.999,1.028)
Stage	0.783			0.208			0.059		
Primary surgical cytoreduction ^c^	Not significant			0.047	0.427	(0.185,0.987)	0.00002	0.419	(0.290,0.604)

^a^ Each multivariable Cox PH model includes patient age, tumor stage, and primary surgical cytoreduction status. ^b^ Immune signatures were standardized to more easily compare the hazard ratio estimates (HR Est.). ^c^ The primary surgical cytoreduction status HR Est. and confidence interval (CI) values are shown for Optimal vs. Sub-optimal cytoreduction when *p* < 0.10. Otherwise, when primary surgical cytoreduction has *p* > 0.10, the cytoreduction status variable is not included in the multivariable model. ^d^ The results for multivariable Cox PH models using M2TAM, patient age, and tumor stage are not shown as M2TAM had *p* > 0.10 for each cohort.

**Table 3:** Validation of pan cancer-derived immune signatures in TCGA cohorts for uterine corpus endometrial cancer (UCEC ^a^) and high tumor mutational burden (Hi-TMB) breast cancer (BRCA ^a^). Immune signatures were standardized to more easily compare the hazard ratios (HR).

Multivariable Cox. Proportional Hazards Models ^b^	TCGA UCEC Cohort			TCGA Hi-TMB ^c^ BRCA Cohort		
	(OS ^e^, *n* = 370)			(OS ^e^, *n* = 194)		
	*p*-Value	HR ^d^ Est.	HR 95% CI	*p*-Value	HR ^d^ Est.	HR 95% CI
**CLIS**	0.001	0.633	(0.480,0.836)	0.002	0.397	(0.224,0.705)
Age	0.002	1.043	(1.016,1.071)	0.004	1.055	(1.017,1.094)
Stage	<0.00001			0.00001		
PR Status	N/A			0.011	3.375	(1.350,10.437)
**TCT**	0.036	0.733	(0.548,0.980)	0.032	0.521	(0.287,0.944)
Age	0.0005	1.047	(1.020,1.074)	0.005	1.055	(1.016,1.094)
Stage	<0.00001			0.00001		
PR Status	N/A			0.017	3.344	(1.241,9.016)

^a^ Patient characteristics for each cohort are shown in Table S4. ^b^ Multivariable Cox proportional hazards (PH) modeling results for CLIS and TCT immune signatures are in the presence of patient age, tumor stage, for both cohorts and in the presence of progesterone receptor (PR) status for the BRCA-related cohort. ^c^ Hi-TMB was defined as TMB > 2 nonsynonymous somatic variants per Mb. ^d^ Immune signatures were standardized to more easily compare the hazard ratios. ^e^ Overall survival (OS).

## Supplementary Materials

The following are available online at https://www.mdpi.com/2072-6694/12/3/620/s1, Figure S1: The color coding and detailed description of the LM22 immune classes (36) for Figure 2, Figure S2: Hazard ratio (HR) estimates (■) with 95% confidence intervals for individual immune signatures in the multivariable Cox proportional hazards (PH) models from three high-grade serous ovarian cancer (HGSOC) cohorts shown in Table 2. Immune signatures were standardized to more easily compare hazard ratios. Each multivariable Cox models includes patient age and tumor stage. All immune-related signatures shown here have higher levels associated with reduced risk and thus better outcomes, Table S1: The Cancer Genome Atlas (TCGA) Solid Tumor Types; Table S2: Multivariable Cox proportional hazards (PH) models from The Cancer Genome Atlas (TCGA) high-grade serous ovarian cancer (HGSOC) cohort with *BRCA1/2* somatic mutation (Som-Mut) versus wild type (WT) status with immune signatures, patient age, and tumor stage for disease-free survival (DFS). Primary cytoreductive surgery (optimal or sub-optimal) was not used since it was not statistically significant for TCGA DFS by itself or with any of the multivariable models shown below, Table S3: Multivariable Cox proportional hazards (PH) models from The Cancer Genome Atlas (TCGA) and Cleveland Clinic-Charité high-grade serous ovarian cancer (HGSOC) cohort with *COL2A1* status (High or Low expression) with immune signatures, patient age, tumor stage, and primary cytoreduction status for disease-free survival (DFS), Table S4: Patient characteristics for uterine corpus endometrial cancer (UCEC) and high tumor mutational burden (Hi-TMB) breast cancer (BRCA) cohorts in TCGA.

## References

[1] Recurrence. https://ocrfa.org/patients/about-ovarian-cancer/recurrence/.

[2] Chitale R. (2009). Monitoring ovarian cancer: CA125 trial stirs controversy. J. Natl. Cancer Inst..

[3] Hoppenot C., Eckert M.A., Tienda S.M., Lengyel E. (2018). Who are the long-term survivors of high grade serous ovarian cancer?. Gynecol. Oncol..

[4] Verhaak R.G., Tamayo P., Yang J.Y., Hubbard D., Zhang H., Creighton C.J., Fereday S., Lawrence M., Carter S.L., Mermel C.H. (2012). Prognostically relevant gene signatures of high-grade serous ovarian carcinoma. J. Clin. Investig..

[5] Bristow R.E., Tomacruz R.S., Armstrong D.K., Trimble E.L., Montz F.J. (2002). Survival effect of maximal cytoreductive surgery for advanced ovarian carcinoma during the platinum era: A meta-analysis. J. Clin. Oncol..

[6] Aluloski I., Tanturovski M., Jovanovic R., Kostadinova-Kunovska S., Petrusevska G., Stojkovski I., Petreska B. (2017). Survival of Advanced Stage High-Grade Serous Ovarian Cancer Patients in the Republic of Macedonia. Open Access Maced. J. Med Sci..

[7] Block M.S., Kalli K.R., Vierkant R.A., Chen W., Fogarty Z.C., Gentry-Maharaj A., Toloczko A., Hein A., Bouligny A.L., Ovarian Tumor Tissue Analysis (OTTA) Consortium Goode, E.L. (2017). Dose-response association of CD8+ tumor-infiltrating lymphocytes and survival time in high-grade serous ovarian cancer. JAMA Oncol..

[8] Cancer Genome Atlas Research Network (2011). Integrated genomic analyses of ovarian carcinoma. Nature.

[9] Ledermann J., Harter P., Gourley C., Friedlander M., Vergote I.G., Rustin G., Scott C.L., Meier W., Shapira-Frommer R., Safra T. (2014). Olaparib maintenance therapy in patients with platinum-sensitive relapsed serous ovarian cancer: A preplanned retrospective analysis of outcomes by BRCA status in a randomised phase 2 trial. Lancet Oncol..

[10] Pujade-Lauraine E., Ledermann J.A., Selle F., Gebski V., Penson R.T., Oza A.M., Korach J., Huzarski T., Poveda A., Pignata S. (2017). Olaparib tablets as maintenance therapy in patients with platinum-sensitive, relapsed ovarian cancer and a BRCA1/2 mutation (SOLO2/ENGOT-Ov21): A double-blind, randomised, placebo-controlled, phase 3 trial. Lancet Oncol..

[11] Zhang L., Conejo-Garcia J.R., Katsaros D., Gimotty P.A., Massobrio M., Regnani M., Makrigiannakis A., Gray H., Schlienger K., Liebman M.N. (2003). Intratumoral T cells, recurrence, and survival in epithelial ovarian cancer. N. Engl. J. Med..

[12] Sato E., Olson S.H., Ahn J., Bundy B., Nishikawa H., Qian F., Jungbluth A.A., Frosina D., Gnjatic S., Ambrosone C. (2005). Intraepithelial CD8+ tumor-infiltrating lymphocytes and a high CD8+/regulatory T cell ratio are associated with favorable prognosis in ovarian cancer. Proc. Natl. Acad. Sci. USA.

[13] Kandalaft L.E., Odunsi K., Coukos G. (2019). Immunotherapy in Ovarian Cancer: Are We There Yet?. J. Clin. Oncol..

[14] Zhang M., He Y., Sun X., Li Q., Wang W., Zhao A., Di W. (2014). A high M1/M2 ratio of tumor-associated macrophages is associated with extended survival in ovarian cancer patients. J. Ovarian Res..

[15] Taub D.D., Lloyd A.R., Conlon K., Wang J.M., Ortaldo J.R., Harada A., Matsushima K., Kelvin D.J., Oppenheim J.J. (1993). Recombinant human interferon-inducible protein 10 is a chemoattractant for human monocytes and T lymphocytes and promotes T cell adhesion to endothelial cells. J. Exp. Med..

[16] Groom J.R., Luster A.D. (2011). CXCR3 in T cell function. Exp. Cell Res..

[17] Slaney C.Y., Kershaw M.H., Darcy P.K. (2014). Trafficking of T cells into tumors. Cancer Res..

[18] Abron J.D., Singh N.P., Murphy A.E., Mishra M.K., Price R.L., Nagarkatti M., Nagarkatti P.S., Singh U.P. (2018). Differential role of CXCR3 in inflammation and colorectal cancer. Oncotarget.

[19] Bronger H., Singer J., Windmüller C., Reuning U., Zech D., Delbridge C., Dorn J., Kiechle M., Schmalfeldt B., Schmitt M. (2016). CXCL9 and CXCL10 predict survival and are regulated by cyclooxygenase inhibition in advanced serous ovarian cancer. Br. J. Cancer.

[20] Lieber S., Reinartz S., Raifer H., Finkernagel F., Dreyer T., Bronger H., Jansen J.M., Wagner U., Worzfeld T., Muller R. (2018). Prognosis of ovarian cancer is associated with effector memory CD8+ T cell accumulation in ascites, CXCL9 levels and activation-triggered signal transduction in T cells. Oncoimmunology.

[21] Iglesia M.D., Parker J.S., Hoadley K.A., Serody J.S., Perou C.M., Vincent B.G. (2016). Genomic analysis of immune cell infiltrates across 11 tumor types. JNCI: J. Natl. Cancer Inst..

[22] Konecny G.E., Wang C., Hamidi H., Winterhoff B., Kalli K.R., Dering J., Ginther C., Chen H.-W., Dowdy S., Cliby W. (2014). Prognostic andtherapeutic relevance of molecular subtypes in high-grade serous ovarian cancer. JNCI: J. Natl. Cancer Inst..

[23] Ganapathi M.K., Jones W.D., Sehouli J., Michener C.M., Braicu I.E., Norris E.J., Biscotti C.V., Vaziri S.A.J., Ganapathi R.N. (2016). Expression profile of COL2A1 and the pseudogene SLC6A10P predicts tumor recurrence in high-grade serous ovarian cancer. Int. J. Cancer.

[24] Thomas A., Routh E.D., Pullikuth A., Jin G., Su J., Chou J.W., Hoadley K.A., Print C., Knowlton N., Black M.A. (2018). Tumor mutational burden is a determinant of immune-mediated survival in breast cancer. Oncoimmunology.

[25] Ellrott K., Bailey M.H., Saksena G., Covington K.R., Kandoth C., Stewart C., Hess J., Ma S., Chiotti K.E., McLellan M. (2018). Scalable open science approach for mutation calling of tumor exomes using multiple genomic pipelines. Cell Syst..

[26] Krishnan V., Berek J.S., Dorigo O. (2017). Immunotherapy in ovarian cancer. Curr. Probl. Cancer.

[27] Peranzoni E., Lemoine J., Vimeux L., Feuillet V., Barrin S., Kantari-Mimoun C., Bercovici N., Guérin M., Biton J., Ouakrim H. (2018). Macrophages impede CD8 T cells from reaching tumor cells and limit the efficacy of anti–PD- 1 treatment. Proc. Natl. Acad. Sci. USA.

[28] (2018). Powerful combination therapies (editorial). Nat. Biomed. Eng..

[29] Böhm S., Montfort A., Pearce O.M., Topping J., Chakravarty P., Everitt G.L., Clear A., McDermott J.R., Ennis D., Dowe T. (2016). Neoadjuvant chemotherapy modulates the immune microenvironment in metastases of tubo-ovarian high-grade serous carcinoma. Clin. Cancer Res..

[30] Lo C.S., Sanii S., Kroeger D.R., Milne K., Talhouk A., Chiu D.S., Rahimi K., Shaw P.A., Clarke B.A., Nelson B.H. (2017). Neoadjuvant chemotherapy of ovarian cancer results in three patterns of tumor-infiltrating lymphocyte response with distinct implications for immunotherapy. Clin. Cancer Res..

[31] Thorsson V., Gibbs D.L., Brown S.D., Wolf D., Bortone D.S., Yang T.H., Porta-Pardo E., Gao G.F., Plaiser C.L., Eddy J.A. (2018). The immune landscape of cancer. Immunity.

[32] Aran D., Hu Z., Butte A.J. (2017). xCell: Digitally portraying the tissue cellular heterogeneity landscape. Genome Biol..

[33] Bastaki S., Irandoust M., Ahmadi A., Hojjat-Farsangi M., Ambrose P., Hallaj S., Edalati M., Ghalamfarsa G., Azizi G., Yousefi M. (2020). PD-L1/PD-1 axis as a potent therapeutic target in breast cancer. Life Sci..

[34] Fan C.A., Reader J., Roque D.M. (2018). Review of Immune Therapies Targeting Ovarian Cancer. Curr. Treat. Options Oncol..

[35] Puzzoni M., Silvestris N., Leone F., Giampieri R., Faloppi L., Demurtas L., Dell’Aquila E., Marino D., Brunetti O., Garattini S.K. (2016). The immune revolution in gastrointestinal tumours: Leading the way or just following?. Target. Oncol..

[36] Newman A.M., Liu C.L., Green M.R., Gentles A.J., Feng W., Xu Y., Hoang C.D., Diehn M., Alizadeh A.A. (2015). Robust enumeration of cell subsets from tissue expression profiles. Nat. Methods.

[37] Rőszer T. (2015). Understanding the mysterious M2 macrophage through activation markers and effector mechanisms. Mediat. Inflammatio.

[38] Bindea G., Mlecnik B., Tosolini M., Kirilovsky A., Waldner M., Obenauf A.C., Angell H., Fredriksen T., Lafontaine L., Berger A. (2013). Spatiotemporal dynamics of intratumoral immune cells reveal the immune landscape in human cancer. Immunity.

[39] Li B., Dewey C.N. (2011). RSEM: accurate transcript quantification from RNA-Seq data with or without a reference genome. BMC bioinformatics.

[40] Broad Institute TCGA Genome Data Analysis Center: Analysis-ready standardized TCGA data from Broad GDAC Firehose 2016_01_28 run (2016). Broad Institute of MIT and Harvard. Dataset.

