# Supplementary material for: RNA Immune Signatures from Pan-Cancer Analysis Are Prognostic for High-Grade Serous Ovarian Cancer and Other Female Cancers
Source: Cancers (Basel). 2020 Mar 7;12(3):620. doi: 10.3390/cancers12030620 (PMC7139955; doi:10.3390/cancers12030620)
Supplement: Supplementary file 1 [file cancers-12-00620-s001.pdf]

Supplementary Materials

RNA Immune Signatures from Pan-Cancer Analysis are Prognostic for High Grade Serous Ovarian Cancer and Other Female Cancers

Wendell D. Jones, Chad M. Michener, Charles Biscotti, Iona Braicu, Jalid Sehouli, Mahrukh K. Ganapathi and Ram N. Ganapathi

B cells naive	Monocytes
B cells memory	Macrophages M0
Plasma cells	Macrophages M1
T cells CD8	Macrophages M2
T cells CD4 memory activated	Dendritic cells resting
T cells CD4 naive	Dendritic cells activated
T cells CD4 memory resting	Mast cells resting
T cells follicular helper	Mast cells activated
T cells regulatory (Tregs)	Eosinophils
T cells gamma delta	Neutrophils
NK cells resting	
NK cells activated	

Figure S1. The color coding and detailed description of the LM22 immune classes (36) for Figure 2.

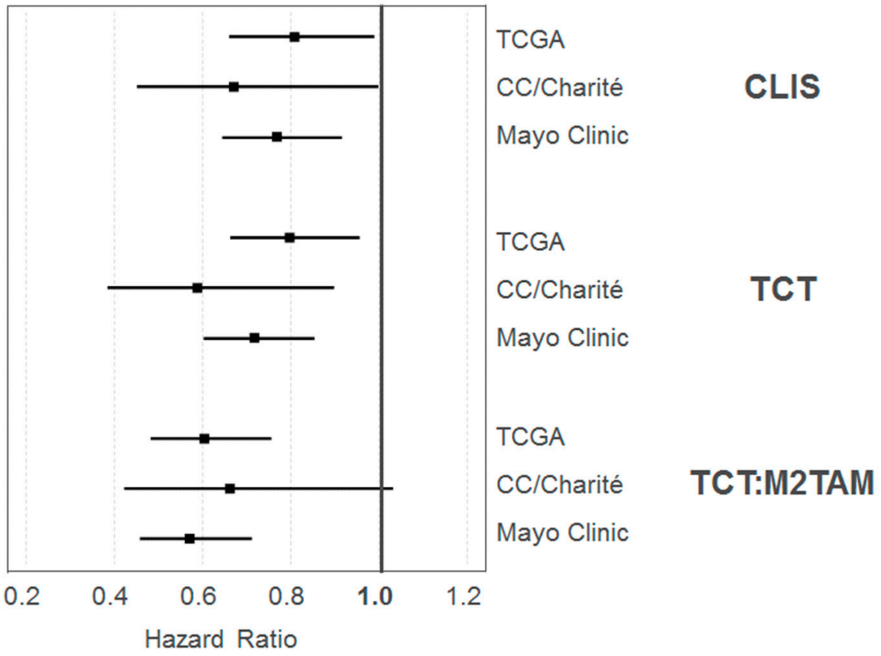

Figure S2. Hazard ratio (HR) estimates (■) with 95% confidence intervals for individual immune signatures in the multivariable Cox proportional hazards (PH) models from three high grade serous ovarian cancer (HGSOC) cohorts shown in Table 2. Immune signatures were standardized to more easily compare hazard ratios. Each multivariable Cox models includes patient age and tumor stage (and primary cytoreductive surgery status when significant). All immune-related signatures shown here have higher levels associated with reduced risk and thus better outcomes.

Table S1. The Cancer Genome Atlas (TCGA) Solid Tumor Types.

1	ACC - Adrenocortical Carcinoma
2	BLCA - Bladder Urothelial Carcinoma
3	BRCA – Breast Invasive Carcinoma
4	CESC - Cervical Squamous Cell Carcinoma and Endocervical Adenocarcinoma
5	COADREAD [a] - Colon Adenocarcinoma (COAD), Rectum Adenocarcinoma (READ)
6	ESCA- Esophageal Carcinoma
7	GBM - Glioblastoma Multiforme
8	HNSC - Head and Neck Squamous Cell Carcinoma
9	KICH - Kidney Chromophobe
10	KIRC - Kidney Renal Clear Cell Carcinoma
11	KIRP - Kidney Renal Papillary Cell Carcinoma
12	LGG - Brain Lower Grade Glioma
13	LIHC - Liver Hepatocellular Carcinoma
14	LUNG [a] - Lung Adenocarcinoma (LUAD), Lung Squamous Cell Carcinoma (LUSC)
15	MESO - Mesothelioma
16	OV - Ovarian Serous Cystadenocarcinoma
17	PAAD - Pancreatic Adenocarcinoma
18	PCPG - Pheochromocytoma and Paraganglioma
19	PRAD - Prostate Adenocarcinoma
20	SARC - Sarcoma
21	SKCM - Skin Cutaneous Melanoma
22	STAD - Stomach Adenocarcinoma
23	TGCT - Testicular Germ Cell Tumors
24	THCA - Thyroid Carcinoma
25	THYM - Thymoma
26	UCEC - Uterine Corpus Endometrial Carcinoma
27	UCS - Uterine Carcinosarcoma

[a] In Figure 2 for the purpose of visualization only, the TCGA identifiers LUAD and LUSC types have been combined into one identifier (LUNG), and COAD and READ identifiers have been combined into one identifier (COADREAD). Thus, 29 original types are coded into 27 groups.

Table S2. Multivariable Cox proportional hazards (PH) models from The Cancer Genome Atlas (TCGA) high grade serous ovarian cancer (HGSOC) cohort with *BRCA1/2* somatic mutation (Som-Mut) versus wild type (WT) status with immune signatures, patient age, and tumor stage for disease-free survival (DFS). Primary cytoreductive surgery (optimal or sub-optimal) was not used since it was not statistically significant for TCGA DFS by itself or with any of the multivariable models shown below.

**Multivariable Cox PH Models**	**TCGA (DFS)**		
	***p*-Value**	**HR Est.**	**HR 95% Conf. Interval**
**CLIS** [a]	0.009	0.792	(0.665,0.944)
Age	0.020	1.019	(1.003,1.036)
Stage	0.390		
BRCA1/2 Som-Mut	0.027	0.384	(0.164,0.897)
**TCT** [b]	0.012	0.813	(0.692,0.955)
Age	0.030	1.018	(1.002,1.034)
Stage	0.602		
BRCA1/2 Som-Mut	0.024	0.379	(0.163,0.882)
**TCT:M2TAM** [c]	0.0002	0.659	(0.530,0.821)
Age	0.151	1.012	(0.996,1.028)
Stage	0.551		
BRCA1/2 Som-Mut	0.028	0.387	(0.165,0.905)

[a] *p*-value for CLIS when the *BRCA1/2* Som-Mut factor is not included is 0.01; [b] *p*-value for TCT when the *BRCA1/2* Som-Mut factor is not included is 0.015; [c] *p*-value for TCT:M2TAM when the *BRCA1/2* Som-Mut factor is not included is 0.0002.

Table S3. Multivariable Cox proportional hazards (PH) models from The Cancer Genome Atlas (TCGA) and Cleveland Clinic-Charité high grade serous ovarian cancer (HGSOC) cohort with *COL2A1* status (High or Low expression) with immune signatures, patient age, tumor stage, and primary cytoreduction status for disease-free survival (DFS).

**Multivariable Cox PH Models**	**TCGA (DFS)**			**Cleveland Clinic-Charité (DFS)**		
	***p*-value**	**HR Est.**	**HR 95% Conf Interval**	***p*-value**	**HR Est.**	**HR 95% Conf Interval**
**CLIS** [a]	0.0034	0.761	(0.634,0.914)	0.0004	0.458	(0.297,0.708)
Age	0.047	1.016	(1.000,1.032)	0.306	1.015	(0.986,1.045)
Stage	0.641			0.072		
COL2A1 High vs Low [c]	0.044	0.704	(0.500,0.991)	0.013	0.458	(0.297,0.708)
Primary cytoreduction (optimal or sub-optimal) [d]	Not significant			0.023	0.397	(0.179,0.794)
**TCT** [b]	0.0036	0.779	(0.658,0.922)	0.0011	0.490	(0.319,0.752)
Age	0.072	1.014	(0.999,1.030)	0.305	1.016	(0.985,1.048)
Stage	0.873			0.060		
COL2A1 High vs Low [c]	0.033	0.686	(0.495,0.971)	0.027	0.404	(0.181,0.902)
Primary cytoreduction (optimal or sub-optimal) [d]	Not significant			0.013	0.368	(0.167,0.811)

[a] *p*-value for Cytotoxic Lymphocyte Immune Signature (CLIS) when the *COL2A1* factor is not included is 0.01 and 0.003 for TCGA and Cleveland Clinic-Charité cohorts respectively; [b] *p*-value for T-cell trafficking (TCT) when the COL2A1 factor is not included is 0.015 and 0.007 for TCGA and Cleveland Clinic-Charité cohorts respectively; [c] *COL2A1* High expression was defined as being above the median expression level in the cohort; [d] Primary surgical cytoreduction (optimal or sub-optimal) was not significant either by itself or in the presence of other co-variables for the TCGA cohort and thus was not used in the model.

Table S4. Patient characteristics for uterine corpus endometrial cancer (UCEC) and high tumor mutational burden (Hi-TMB) breast cancer (BRCA) cohorts in TCGA.

**Cohort Characteristics**	**UCEC**	**Hi-TMB BRCA**
No. of patients (*n*)	370	194
n with $t \leq$ 1-year (censored)[a]	48 (31)	36 (32)
n with 1 year < $t \leq$ 5-year (censored)[a]	226 (191)	118 (106)
n with $t >$ 5-year (censored)[a]	96 (89)	40 (35)
% patients w/ censored survival	84%	89%
min % with ≥ 5-year survival	39%	21%
Stage ≤ 2	272 (74%)	152 (78%)
Stage 3	79 (21%)	39 (20%)
Stage 4	19 (5%)	3 (2%)
Median Age (years)	63	61
Interquartile Range of Age (years)	(57, 77)	(52, 71)
ER[b] Status (% positive)	NA	65%
PR[b] Status (% positive)	NA	52%
RNA measurement platform	RNA-Seq	RNA-Seq

[a] Survival time (OS) is represented by "*t*"; [b] ER, estrogen receptor; PR, progesterone receptor.

© 2020 by the authors. Licensee MDPI, Basel, Switzerland. This article is an open access article distributed under the terms and conditions of the Creative Commons Attribution (CC BY) license (http://creativecommons.org/licenses/by/4.0/).
